# Physical exercise and loneliness in higher education: a national study of Norwegian students

**DOI:** 10.1186/s40359-026-04183-4

**Published:** 2026-02-14

**Authors:** Silje Blindheim, Tone Langjordet Johnsen, Michael Grasdalsmoen, Børge Sivertsen, Hege R. Eriksen

**Affiliations:** 1 Department of Sport, Food and Natural Sciences, Western Norway University of Applied Sciences, Inndalsveien 28, Bergen, 5063 Norway; 2 Department of Health, Social and Welfare Studies, University of South-Eastern Norway, Horten, Norway; 3 Department of Health Promotion, Norwegian Institute of Public Health, Bergen, Norway; 4 Department of Research & Innovation, Helse Fonna HF, Haugesund, Norway

**Keywords:** University students, Mental health, Loneliness, Physical activity, Physical exercise

## Abstract

**Background:**

Physical activity has been associated with lower levels of loneliness. However, limited research has explored whether exercise frequency, intensity, and duration are associated with loneliness. The aim of this study was to investigate the associations between exercise frequency, intensity, and duration, and loneliness among Norwegian college and university students.

**Methods:**

Data from the National Student Health and Well-being Survey in Norway (SHoT 2022), including 59,544 full-time students aged 18–35 years, were analyzed. The Three-Item Loneliness Scale was used to measure three dimensions of loneliness: lack of companionship, feeling left out, and feeling isolated. Self-reported physical exercise was categorized by frequency, intensity, and duration. Logistic regression was applied to examine the association between physical exercise and loneliness, adjusting for sociodemographic (age, relationship status, and parents’ educational level) and behavioral (sleep duration, smoking, snus [smokeless tobacco], and alcohol consumption) factors.

**Results:**

Physical exercise was inversely associated with loneliness across all dimensions. The strongest associations were observed between exercise frequency and feeling isolated. In sex-stratified analyses, associations were generally stronger in males than in females for higher exercise frequency and longer duration. Specifically, in adjusted models, females exercising 4 to 5 times per week had lower odds of feeling isolated than non-exercisers (OR 0.43), whereas males exercising almost daily also had lower odds of feeling isolated (OR 0.47). Moderate-intensity exercise was associated with lower loneliness levels for both sexes (ORs 0.65–0.76), compared with low-intensity exercise.

**Conclusion:**

Regular physical exercise was associated with lower levels of loneliness among college and university students. Higher exercise frequency and longer duration showed stronger associations among males than females. Sex-specific, longitudinal, and experimental studies are needed to identify causal relationships and explore underlying mechanisms.

## Background

Loneliness has emerged as a significant public health concern associated with a wide range of adverse physical and mental health outcomes, including higher risk of depression, anxiety, suicidal ideation [1], cardiovascular heart disease [2], and mortality [3]. Loneliness is commonly defined as a subjective feeling of non-belonging, often characterized by a perceived deficiency in the quantity or, in particular, the quality of social relationships [1]. While much research has focused on loneliness among older adults, less attention has been paid to loneliness among young adults, particularly university students [4–6]. The COVID-19 pandemic increased the focus on this population due to higher reports of loneliness [6–8]. A significant rise in loneliness among Norwegian university and college students was observed between 2014 (16.5%) and 2018 (23.6%), with the proportion of males feeling “extremely” lonely more than doubling [5]. Loneliness continued to rise substantially in both sexes between 2018 (23.6%) and 2021 (31.9%). Although declining to 28.2% in 2022 (post-pandemic), loneliness levels in 2022 remained higher than pre-pandemic levels [6].

For those experiencing the transition from adolescence to young adulthood, university students may be particularly vulnerable to lonelness. This susceptibility arises from factors such as the challenges of becoming independent, forming new social networks, and adapting to new schedules [9]. Sociodemographic factors, including sex, may also influence loneliness, although findings remain inconsistent. Hysing et al. [5] found that being female, single, and living alone significantly contributed to loneliness among Norwegian students, with the youngest (18–20 years) and the oldest (26–35 years) students reporting the highest levels of loneliness. In contrast, Wenig et al. [4] found no associations between loneliness, gender, or age in German university students. Behavioral factors may also play a role in loneliness. For instance, risky behaviors such as smoking [10] and excessive alcohol consumption [7] have been linked to loneliness among young adults.

Cross-sectional studies have indicated that physical activity, defined as any bodily movement produced by skeletal muscles that results in energy expenditure [11], is associated with lower levels of loneliness among university students [4, 9]. Additionally, physical exercise, a planned, structured, and repetitive form of physical activity [11], is associated with improved mental health [12] and lower levels of loneliness in this population [13]. There are indications that the quality aspects of physical activity, such as context and opportunities for social interaction, may play a more decisive role in alleviating loneliness than activity levels alone [14, 15]. This aligns with social support theory, which posits that social support is a protective factor against various mental health issues [16]. Within this framework, physical exercise may reduce loneliness by providing access to emotional (e.g., empathy, encouragement), instrumental (e.g., practical help), and informational (e.g., advice, shared knowledge) support [17]. Previous studies involving students have reported positive associations between social support and physical activity [18], suggesting that exercise may be associated with lower loneliness through its relationship with students’ social networks.

However, exercise can provide substantial physiological or psychological benefits even when not performed in a social context [19]. Furthermore, loneliness and physical inactivity often coexist, with loneliness itself potentially diminishing motivation for physical activity and social engagement, indicating a complex bidirectional relationship [20].

Initiatives to alleviate loneliness among young adults are needed [6]. Physical exercise is a low-cost intervention with minimal side effects and has shown promising benefits for mental health outcomes in young people [21]. Evidence indicates a dose–response relationship where higher frequency, intensity, and duration of physical exercise are associated with lower levels of negative mental health outcomes such as anxiety, depression, and suicidal ideation among students [12]. However, little is known about how exercise patterns, including frequency, intensity, and duration, are related to loneliness among students in higher education.

Using a large-scale dataset from the Students’ Health and Well-being Study (SHoT 2022), we explored the associations between exercise frequency, intensity, duration, and loneliness among Norwegian college and university students while accounting for sociodemographic and behavioral factors. We hypothesized that higher levels of exercise frequency, intensity, and duration are associated with lower levels of loneliness.

## Methods

### Procedure

The SHoT study is a national survey conducted every fourth year among full-time higher education students in Norway, initiated and funded by the three largest student welfare organizations: SiO, Sammen, and SiT. The primary goal of the survey is to assess students’ self-reported health, well-being, quality of life, and psychosocial environment, as well as study-related factors. These data are used to identify health determinants, examine trends, and inform targeted interventions to enhance students’ health and well-being. Self-report questionnaires facilitate cost-efficient, large-scale data collection from diverse student populations and institutions. More information on the SHoT survey has been previously provided [22].

This study was conducted between February and April 2022. Full-time Norwegian students aged between 18 and 35 years and pursuing higher education in Norway or abroad were invited to participate. Invitations were sent via email and SMS, followed by two additional email reminders and one SMS reminder. Awareness was further promoted through social media campaigns, outreach by educational institutions, posters, and digital advertisements on campuses. A total of 169,572 students were invited to participate, and 59,544 completed the online questionnaire (response rate of 35.1%) [23].

### Dependent variables

#### Loneliness

Loneliness was assessed using the Three-Item Loneliness Scale (T-ILS) [24]. This is a shortened version of the UCLA Loneliness Scale [25], one of the most widely used instruments for measuring subjective experiences of loneliness in various populations, including college and university students [26]. The T-ILS consists of three questions measuring how often the respondent felt lonely during the past year: 1) “How often do you feel that you lack companionship?” 2) “How often do you feel left out?” and 3) “How often do you feel isolated from others?” Questions are rated on a 5-point Likert scale: “never,” “seldom,” “sometimes,” “often,” or “very often.” The T-ILS has demonstrated satisfactory reliability and both convergent and discriminant validity [24, 26]. In line with a study by Hysing et al. [5], each T-ILS item was dichotomized: the response categories “often” and “very often” were coded as 1 (indicating loneliness), and the response categories “never,” “seldom,” and “sometimes” were coded as 0. This approach was chosen to ensure comparability with previous publications on SHoT and to facilitate clear interpretation in logistic regression analyses, although we acknowledge that it reduces the granularity of the data. The complete distribution of the SHoT 2022 T-ILS has been published elsewhere [6].

### Independent variables

#### Physical exercise

Before the participants could answer the questions about physical exercise, they were presented with a brief definition: “By exercise we mean that you, for example, go for a walk, go skiing, swim, or take part in a sport.” Physical exercise patterns were measured using three questions assessing average weekly exercise frequency, intensity, and duration. The questions included: 1) Frequency: “In an average week, how frequently do you perform physical exercise?” with the response categories “never,” “less than once a week,” “once a week,” “2–3 times per week,” “4–5 times per week,” or “almost every day”; 2) Intensity: “If you perform physical exercise as frequently as once or more times a week, how hard do you push yourself?” with the response categories “I take it easy without breaking into a sweat or losing my breath,” “I push myself so hard that I lose my breath and break into a sweat,” or “I push myself to near exhaustion”; and 3) Duration: “How long does each session last?” with the response categories “less than 15 min,” “15–29 min,” “30 min to 1 h,” or “more than 1 h.” These questions have been used in prior SHoT studies [12, 27, 28] and in the large population-based Nord-Trøndelag Health Study (HUNT), demonstrating acceptable reliability and validity [29, 30].

### Control variables

#### Sociodemographic factors

Information regarding participants’ age and sex was derived from their 11-digit Norwegian national identity numbers. In the analysis, age was treated as a categorical variable using the following categories: 18–20 years, 21–22 years, 23–25 years, 26–28 years, and 29–35 years. Participants were also asked about their relationship status (single vs. in a relationship) and their parents’ educational level (primary school, upper secondary school, or college/university). In addition, they were asked whether they or their parents were born outside Norway.

### Behavioral factors

#### Sleep duration

Study participants indicated their usual sleep patterns by specifying their bedtime and rise time, measured in hours and minutes. This information was recorded separately for weekdays and weekends. The average daily sleep duration was categorized into five groups and used as a categorical variable: 1 = < 6 h, 2 = 6–6:59 h, 3 = 7–7:59 h, 4 = 8–8:59 h, and 5 = 9 + h. Further details regarding the measurement of sleep patterns in the SHoT surveys are available in a previous publication [31].

#### Tobacco/Snus

Smoking and snus use were assessed using the following questions: “Do you smoke?” and “Do you use snus or something similar?” The response options were “yes, daily,” “yes, occasionally,” or “no.” A dichotomous variable, “daily smoking,” was defined as daily smoking versus either occasional or no smoking. Similarly, a dichotomous variable for “daily snus use” was created (see [32] for comparative norms).

#### Alcohol-related problems

Potential alcohol-related problems were assessed using the Alcohol Use Disorders Identification Test (AUDIT), a widely accepted instrument developed by the World Health Organization for detecting risky or harmful alcohol consumption over the previous 12 months [33, 34]. The 10-item AUDIT measures three domains: alcohol consumption (items 1–3), alcohol dependence (items 4–6), and alcohol-related problems (items 7–10) [33]. AUDIT scores range from 0 to 40, with the original guidelines proposing the following cut-off scores: 0–7 (no or low risk), 8–15 (risky alcohol use), 16–19 (harmful alcohol use), and 20–40 (dependent alcohol use) [34]. In this study, we categorized participants as follows: 1 = no/low-risk alcohol use, 2 = problem-related alcohol use (including both risky and harmful alcohol use), and 3 = dependent alcohol use. More details regarding the AUDIT inventory used in the SHoT surveys can be found in a previous publication [35].

### Ethics

The study adhered to the Helsinki Declaration [36] and was approved by the Regional Committee for Medical and Health Research Ethics in Western Norway (SHoT 2022, no. 326437). Participants provided written informed consent after receiving a comprehensive description of the study.

### Statistics

Data were analyzed using IBM SPSS Statistics Version 29 (SPSS Inc., Chicago, IL, USA) for Windows. Descriptive statistics, including prevalence rates, were used to describe sociodemographic and behavioral factors, loneliness, and physical exercise. Logistic regression analyses were conducted using the three loneliness dimensions as dependent variables. Interaction terms between sex and physical exercise were tested first. Subsequently, separate analyses for males and females (sex-stratified models) were conducted to examine the associations between physical exercise patterns (frequency, intensity, and duration) and dimensions of loneliness (lack of companionship, feeling left out, and feelings of isolation). The initial models were unadjusted, whereas the adjusted models accounted for sociodemographic and behavioral factors. Effect-size estimates are presented as odds ratios (ORs) with 95% confidence intervals (CIs) indicating the associations between physical exercise and the likelihood of reporting loneliness. The Wald test was used to identify significant predictors, and multicollinearity among the independent variables was assessed using variance inflation factors (VIFs). Statistical significance was set at p < 0.05 for all analyses. Considering the large sample size, the low proportion of missing data, and the need to maintain consistency in analyses, participants with missing data were excluded through listwise deletion.

## Results

### Sample characteristics

Among the participants, 66.5% were female and 33.5% were male, with the majority aged between 21 and 25 years (see Table 1). Approximately half were in a relationship (52.1%), and most had college/university-educated parents (63.7% of mothers and 54.8% of fathers). Regarding behavioral factors, 24.8% used snus (smokeless tobacco) and 8% smoked. Most (59.8%) reported sleeping for 7 to 9 h per night. In terms of alcohol use, 56.4% of participants reported no or low-risk alcohol use, 29.1% reported problematic alcohol use, and 14.5% were classified as alcohol dependent. Female consistently reported higher levels of loneliness in all dimensions (see Table 2). About one-third of both male (33%) and female (37%) reported exercising 2–3 times per week. Among those who exercised at least once a week, approximately 69% of both sexes reported exercising at moderate intensity. The majority reported sessions lasting 30 min or longer (88% of males and 86% of females) (see Tables 3 and 4).

**Table 1:** Demographic and behavioral factors among college/university students in SHoT 2022

Demographic variables		Total	Male		Female	
	n	%	n	%	n	%
Gender	59544		19967	33.5	39577	66.5
Age	59541		19966		39575	
18–20	8495	14.3	2312	11.6	6183	15.6
21–22	16214	27.2	5301	26.6	10913	27.6
23–25	17504	29.4	6087	30.5	11417	28.8
26–28	12838	21.6	4806	24.1	8032	20.3
29–35	4490	7.5	1460	7.3	3030	7.7
Relationship status	59544		19967		39577	
Singe	28518	47.9	10362	51.9	18156	45.9
In relationship	31026	52.1	9605	48.1	21421	54.1
Migration background	59544		19967		39577	
Self and/or parent(s) born abroad	6403	10.8	2146	10.7	4257	10.8
Born in Norway	53141	89.2	17821	89.3	35320	89.2
Mothers education level	57366		18952		38414	
Primary school	3615	6.3	1135	5.7	2480	6.5
Upper secondary school	17219	30.0	5301	26.5	11918	31.0
College/university	36532	63.7	12516	62.7	24016	62.5
Fathers education level	56213		18720		37493	
Primary school	4269	7.6	1332	6.7	2937	7.8
Upper secondary school	21145	37.6	6294	33.6	14851	39.6
College/university	30799	54.8	11094	59.3	19705	52.6

Behavioral variables		Total	Male		Female	
	n	%	n	%	n	%
Snus (smokeless tobacco)	58997		19692		39305	
Yes	14660	24.8	5796	29.4	8864	22.6
No	44337	75.2	13896	70.6	30441	77.4
Smoke	59014		19697		39317	
Yes	4742	8.0	2005	10.2	2737	7.0
No	54272	92.0	17692	89.8	36580	93
Sleep duration	55449		18690		36759	
< 6 h	5928	10.7	1956	10.5	3972	10.8
6–6:59 h	10147	18.3	3462	18.5	6685	18.2
7–7:59 h	18344	33.1	6523	34.9	11821	32.2
8–8:59 h	14830	26.7	4907	26.3	9923	27.0
9 + hrs	6200	11.2	1842	9.9	4358	11.9
Alcohol related problems AUDIT	59544		39967		39577	
No/low risk alcohol use	33558	56.4	7747	38.8	25811	65.2
Problem related alcohol use	17356	29.1	7288	36.5	10068	25.4
Dependent alcohol use	8630	14.5	4932	24.7	3698	9.3

**Table 2:** Dichotomous loneliness scores (T-ILS) among college/university students in SHoT 2022

Dichotomous loneliness scores	Total		Male		Female	
	n	%	n	%	n	%
(T-ILS 1) Lack of companionship						
Never, seldom, sometimes	42866	72.8	14700	74.9	28166	71.8
Often, very often	15983	27.2	4935	25.1	11048	28.2
(T-ILS 2) Feel left out						
Never, seldom, sometimes	47252	80.3	16376	83.5	30876	78.8
Often, very often	11556	19.7	3229	16.5	8327	21.2
(T -ILS 3) Feel isolated						
Never, seldom, sometimes	47563	80.9	16177	82.5	31386	80.1
Often, very often	11215	19.1	3425	17.5	7790	19.9

**Table 3:** Association between physical exercise and loneliness among male college/university students in SHoT 2022

			Lack companionship				Feel left out				Feel isolated			
			Unadjusted model		Adjusted model		Unadjusted model		Adjusted model		Unadjusted model		Adjusted model	
Males	n	%	OR	(95% CI)	OR	(95% CI)	OR	(95% CI)	OR	(95% CI)	OR	(95% CI)	OR	(95% CI)
Physical exercise frequency														
Never (ref)	943	4.8	1.00	-	1.00	-	1.00	-	1.00	-	1.00	-	1.00	-
Less than once a week	2018	10.3	0.83	(0.70—0.98)	1.06	(0.87—1.29)	0.79	(0.66—0.94)	0.96	(0.78—1.18)	0.77	(0.65—0.91)	0.94	(0.77—1.15)
Once a week	2221	11.3	0.73	(0.62—0.86)	0.94	(0.77—1.14)	0.60	(0.50—0.72)	0.74	(0.60—0.91)	0.54	(0.45—0.64)	0.67	(0.55—0.82)
2–3 times per week	6497	33.0	0.62	(0.54—0.72)	0.83	(0.69—0.99)	0.50	(0.43—0.58)	0.62	(0.52—0.75)	0.45	(0.38—0.52)	0.58	(0.49—0.70)
4–5 times per week	4643	23.6	0.59	(0.51—0.69)	0.75	(0.62—0.90)	0.44	(0.37—0.52)	0.55	(0.45—0.67)	0.41	(0.35—0.48)	0.52	(0.43—0.63)
Almost every day	3363	17.1	0.57	(0.49—0.67)	0.71	(0.59—0.86)	0.40	(0.33—0.47)	0.51	(0.42—0.63)	0.37	(0.32—0.44)	0.47	(0.39—0.58)
Physical exercise intensity														
I take it easy without breaking into sweat or losing my breath (ref)	3614	25.9	1.00	-	1.00	-	1.00	-	1.00	-	1.00	-	1.00	-
I push myself so hard that I loose my breath and break into sweat	12,679	68.7	0.71	(0.66—0.77)	0.75	(0.68—0.82)	0.59	(0.54—0.65)	0.65	(0.59—0.72)	0.59	(0.54—0.65)	0.65	(0.59—0.72)
I push myself to near exhaustion	2316	5.4	0.76	(0.68—0.86)	0.81	(0.70—0.92)	0.67	(0.58—0.76)	0.76	(0.65—0.88)	0.67	(0.58—0.76)	0.75	(0.65—0.87)
Physical exercise duration														
Less than 15 min (ref)	394	2.1	1.00	-	1.00	-	1.00	-	1.00	-	1.00	-	1.00	-
15–29 min	1819	9.8	0.85	(0.67—1.07)	0.92	(0.70—1.21)	0.98	(0.75—1.27)	1.07	(0.79—1.43)	1.02	(0.79—1.32)	1.12	(0.83—1.48)
30 min to 1 h	7761	41.7	0.68	(0.54—0.84)	0.74	(0.57—0.95)	0.70	(0.55—0.90)	0.78	(0.58—1.02)	0.67	(0.52—0.85)	0.76	(0.58—1.00)
More than 1 h	8653	46.5	0.65	(0.53—0.81)	0.68	(0.52—0.87)	0.56	(0.44—0.72)	0.63	(0.48—0.84)	0.57	(0.45—0.73)	0.64	(0.49—0.84)

All adjusted models include accounting for sociodemographic (age, relationship status, parental education, and ethnicity) and behavioral factors (sleep duration, tobacco, snus, and alcohol-related problems); *CI* Confidence interval

**Table 4:** Association between physical exercise and loneliness among female college/university students in SHoT 2022

			Lack companionship				Feel left out				Feel isolated			
			Unadjusted model		Adjusted model		Unadjusted model		Adjusted model		Unadjusted model		Adjusted model	
Females	n	%	OR	(95% CI)	OR	(95% CI)	OR	(95% CI)	OR	(95% CI)	OR	(95% CI)	OR	(95% CI)
Physical exercise frequency														
Never (ref)	1403	3.6	1.00	-	1.00	-	1.00	-	1.00	-	1.00	-	1.00	-
Less than once a week	3693	9.4	0.87	(0.77—0.99)	0.96	(0.83—1.11)	0.73	(0.64—0.83)	0.79	(0.68—0.92)	0.64	(0.56—0.74)	0.68	(0.59—0.80)
Once a week	4716	12.0	0.74	(0.65—0.84	0.81	(0.70—0.94)	0.56	(0.50—0.64)	0.61	(0.52—0.71)	0.49	(0.43—0.56)	0.53	(0.46—0.62)
2–3 times per week	14580	37.1	0.61	(0.54—0.68)	0.68	(0.60—0.78)	0.48	(0.43—0.54)	0.56	(0.48—0.64)	0.42	(0.37—0.47)	0.48	(0.42—0.53)
4–5 times per week	8896	22.6	0.61	(0.54—0.68)	0.66	(0.58—0.76)	0.43	(0.38—0.49)	0.50	(0.44—0.58)	0.37	(0.32—0.41)	0.43	(0.37—0.49)
Almost every day	5989	15.2	0.64	(0.57—0.73)	0.69	(0.60—0.79)	0.48	(0.43—0.55)	0.54	(0.47—0.63)	0.43	(0.38—0.49)	0.48	(0.41—0.56)
Physical exercise intensity														
I take it easy without breaking into sweat or losing my breath (ref)	9744	25.9	1.00	-	1.00	-	1.00	-	1.00	-	1.00	-	1.00	-
I push myself so hard that I loose my breath and break into sweat	25843	68.7	0.78	(0.74—0.82)	0.76	(0.72—0.81)	0.70	(0.66—0.74)	0.71	(0.67—0.76)	0.65	(0.61—0.68)	0.65	(0.61—0.69)
I push myself to near exhaustion	2019	5.4	0.87	(0.78—0.96)	0.84	0.75—0.95)	0.86	(0.77—0.97)	0.85	(0.75—0.96)	0.80	(0.71—0.90)	0.78	(0.68—0.88)
Physical exercise duration														
Less than 15 min (ref)	756	2.0	1.00	-	1.00	-	1.00	-	1.00	-	1.00	-	1.00	-
15–29 min	4638	12.3	0.71	(0.61—0.83)	0.74	(0.61—0.88)	0.71	(0.60—0.84)	0.72	(0.59—0.86)	0.76	(0.64—0.91)	0.82	(0.67—1.00)
30 min to 1 h	20411	54.3	0.60	(0.52—0.70)	0.62	(0.52—0.74)	0.53	(0.46—0.62)	0.55	(0.46—0.66)	0.59	(0.50—0.69)	0.65	(0.54—0.78)
More than 1 h	11797	31.4	0.66	(0.56—0.76)	0.63	(0.53—0.75)	0.55	(0.47—0.64)	0.55	(0.46—0.66)	0.58	(0.49—0.69)	0.61	(0.51—0.74)

All adjusted models include accounting for sociodemographic (age, relationship status, parental education, and ethnicity) and behavioral factors (sleep duration, tobacco, snus, and alcohol-related problems); *CI* Confidence interval

### Physical exercise and loneliness

#### Sex interactions

Tests of sex × exercise interactions indicated that associations between physical exercise and loneliness varied by sex. Significant interactions were found for exercise frequency (left out, *p =* 0.018; isolated, *p =* 0.006), intensity (left out, *p =* 0.002), and duration (all three loneliness dimensions: left out, *p <* 0.001; isolated, *p <* 0.001; lack of companionship, *p =* 0.012). These findings suggest that the strength and pattern of associations differ for males and females. Therefore, all subsequent results are presented in sex-stratified models.

#### Overall results

**Fig. 1:**
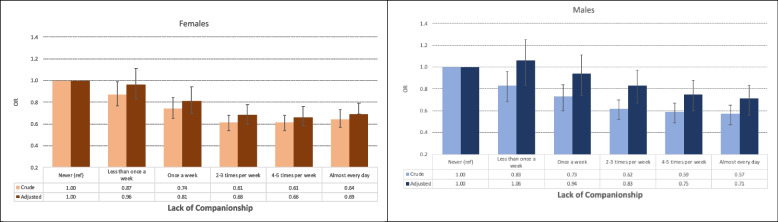
Bar charts illustrate the association between exercise frequency and loneliness, with error bars representing 95% confidence intervals. Adjustments account for sociodemographic (age, relationship status, parental education, ethnicity) and behavioral factors (sleep duration, tobacco use, snus, and alcohol-related problems)

**Fig. 2:**
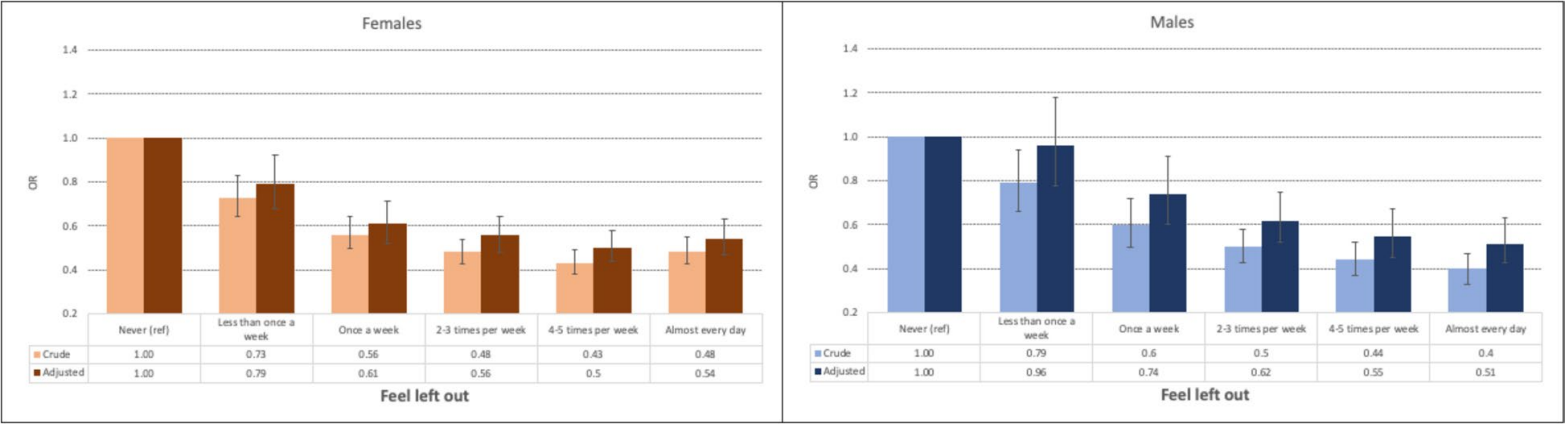
Bar charts illustrate the association between exercise frequency and loneliness, with error bars representing 95% confidence intervals. Adjustments account for sociodemographic (age, relationship status, parental education, ethnicity) and behavioral factors (sleep duration, tobacco use, snus, and alcohol-related problems)

**Fig. 3:**
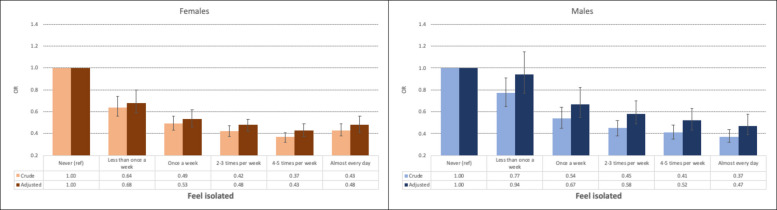
Bar charts illustrate the association between exercise frequency and loneliness, with error bars representing 95% confidence intervals. Adjustments account for sociodemographic (age, relationship status, parental education, ethnicity) and behavioral factors (sleep duration, tobacco use, snus, and alcohol-related problems)

Physical exercise was associated with a lower likelihood of loneliness across all dimensions (see Tables 3 and 4 and Figs. 1, 2 and 3). Generally, higher levels of frequency, intensity, and duration of exercise were associated with lower odds of loneliness, with the most pronounced associations observed at moderate to high exercise levels. The strongest association was found between exercise frequency and feelings of isolation, in both sexes.

##### Exercise frequency

In the unadjusted analyses, females who exercised 4–5 times weekly had the lowest likelihood of loneliness, compared to females who never exercised. The strongest association was observed for feelings of isolation (OR = 0.37, CI: 0.32–0.41, *p <* 0.001), followed by feelings of being left out (OR = 0.43, CI: 0.38–0.49, *p <* 0.001) and lack of companionship (OR = 0.61, CI: 0.54–0.68, *p <* 0.001). After adjusting for sociodemographic and behavioral factors, the associations weakened but remained statistically significant (Table 4 and Figs. 1, 2 and 3).

For males, exercising almost daily was associated with the lowest levels of loneliness, compared to males who never exercised. The strongest association was found for feelings of isolation (OR = 0.37, CI: 0.32–0.44, *p <* 0.001), followed by feelings of being left out (OR = 0.40, CI: 0.33–0.47, *p <* 0.001) and lack of companionship (OR = 0.57, CI: 0.49–0.67, *p <* 0.001). The associations weakened but remained statistically significant in the adjusted analyses (Table 3 and Figs. 1, 2 and 3).

##### Exercise intensity

In the unadjusted analyses, moderate-intensity exercise (i.e., exercising to the point of sweating or breathlessness) was associated with the lowest likelihood of loneliness, compared to low-intensity exercise levels in both sexes. Among females, the strongest associations were found for feeling isolated (OR = 0.65, CI: 0.61–0.68, *p <* 0.001), followed by feeling left out (OR = 0.70, CI: 0.66–0.74, *p <* 0.001), and lack of companionship (OR = 0.78, CI: 0.74–0.82, *p <* 0.001) (Table 4). Among males, the strongest associations were found for feeling left out and isolated (OR = 0.59, CI: 0.54–0.65), followed by a lack of companionship (OR = 0.71, CI: 0.66–0.77, *p <* 0.001) (Table 3). The associations were slightly weakened but remained statistically significant in the adjusted analyses (Tables 3 and 4).

##### Exercise duration

In the unadjusted analyses, female students exercising between 30 min and 1 h were less likely to feel left out (OR = 0.53, CI: 0.46–0.62, *p <* 0.001) or lack companionship (OR = 0.60, CI: 0.52–0.70, *p <* 0.001), compared with females exercising less than 15 min. Females who exercised for more than 1 h had the lowest likelihood of feeling isolated (OR = 0.58, CI: 0.49–0.69, *p <* 0.001). The associations weakened but remained statistically significant in the adjusted analyses (Table 4). Among males, exercising for more than 1 h was most strongly associated with lower loneliness, in all dimensions, compared with males exercising less than 15 min: feeling left out (OR = 0.56, CI: 0.44–0.72, *p <* 0.001), feeling isolated (OR = 0.57, CI: 0.45–0.73, *p <* 0.001), and lack of companionship (OR = 0.65, CI: 0.53–0.81, *p <* 0.001). The associations weakened but remained statistically significant in the adjusted analyses (Table 3).

Across all loneliness dimensions and exercise indicators, unadjusted models explained a limited proportion of variance, with Nagelkerke *R*^2^ values ranging from approximately 0.002 to 0.019. After adjustment for sociodemographic and behavioral factors, the proportion of explained variance increased, with Nagelkerke *R*^2^ values ranging from 0.037 to 0.121.

## Discussion

This nationwide survey, conducted in 2022, identified an inverse association between physical exercise and loneliness among full-time Norwegian college and university students. Generally, higher levels of exercise (including frequency, intensity, and duration) were associated with a lower likelihood of loneliness, with the strongest association observed for feelings of isolation. The associations varied somewhat by sex. Females who exercised 4–5 times per week in sessions lasting 30 min to 1 h reported the lowest likelihood of loneliness. Among males, exercising almost daily and for more than 1 h per session was associated with the lowest odds of loneliness for all dimensions. Moderate-intensity exercise was associated with lowest levels of loneliness in both sexes. After adjusting for sociodemographic and behavioral factors, the association between exercise and loneliness weakened, particularly among males, suggesting that these factors may explain part of the exercise–loneliness relationship.

Consistent with other studies, higher levels of physical exercise were inversely related to loneliness among the students [4, 9, 37, 38]. These findings are furthermore in line with a recent study linking higher exercise levels (frequency, intensity, and duration) to better mental health outcomes (i.e., lower levels of anxiety, depression, and suicidal ideation) among Norwegian college and university students [12]. However, more exercise is not necessarily always better. Although our model did not assess nonlinear associations, Grasdalsmoen et al. [13] found that among athlete students, the protective association between exercise and loneliness plateaued after 7–10 h of weekly exercise. More than 14 h of exercise increased the odds of loneliness, particularly among females. In our study, higher exercise frequency and longer duration were more strongly associated with lower levels of loneliness among males than females. These sex differences align with the findings from the study of Werneck et al. [39], where higher levels of physical activity were associated with a reduced likelihood of social isolation, including feelings of loneliness, among boys. Among girls, moderate activity levels were linked to a lower likelihood of having few friends; however, no association was found with loneliness.

Although our data did not differentiate between exercise types or contexts, participation in exercise may provide a social setting in which support can be generated [15, 37]. Such interactions may explain why higher exercise levels were associated with lower loneliness in our study, as loneliness is commonly described as the subjective experience of lacking meaningful social connections [1]. From the perspective of social support theory [17], social interactions related to exercise may provide emotional, instrumental, and informational support that can help protect against feelings of isolation. At the same time, poor-quality interactions, such as exclusion or criticism, can exacerbate loneliness [40], which indicates that the quality of relationships is as important as their presence [37, 40].

It has been indicated that males have a higher participation rate in sport [41], and have a stronger motivational predisposition toward physical, structured, and competitive activities, which may lead them to engage in team sports more frequently and for longer durations than females [42]. Participation in team sports has been associated with lower loneliness [13, 15] and can enhance perceived social competence through the development of peer relationships fostered by shared goals, collaboration, and time spent with like-minded individuals [15]. In Norway, males are more active in organized sports than females (59.3% vs. 40.7%, including all ages), with football and handball being the most common team sports among both sexes [43]. While females participate in sports, they are more likely to engage in noncompetitive activities such as fitness-oriented or individual exercise [42]. Additionally, females tend to receive more social support related to physical activity, particularly from family and friends [18, 44], and generally possess stronger social-emotional skills than males [44]. These factors may provide females with greater emotional and practical support and facilitate expansion of their social networks beyond the activity itself, potentially accounting for the observed sex differences in exercise patterns and loneliness in our data. These sex-specific exercise participation patterns thus illustrate the complexity of the relationship between physical exercise and loneliness.

Engaging in physical activity may be linked to reduced severity of the negative effects of loneliness, even when the activity is performed alone. Moderate physical activity, such as walking, when integrated into daily routines, has been linked to fewer negative effects of social isolation, potentially benefiting individuals at higher risk for affective disorders, such as those with smaller social networks and higher levels of loneliness [45]. Furthermore, Jennen et al. [46] found that physical activity was associated with lower subsequent loneliness only when it was perceived as enjoyable and when participants felt competent, which underscores the importance of subjective experience of the activity. In addition, the relationship between physical activity and loneliness may be reciprocal [20]. A recent longitudinal study supports the possibility of bidirectional effects, with physical activity having a stronger influence on loneliness than vice versa [47]. However, findings remain mixed [48], which suggests that differences in study designs and measurement approaches complicate comparisons and may obscure potential associations between physical activity and loneliness [47, 48].

After adjusting for sociodemographic and behavioral factors, the association between physical exercise and loneliness remained significant but weakened, particularly among males. Single and socially isolated males have been identified as a high-risk group for loneliness [5]. In addition, male students are more likely to engage in high-risk alcohol consumption, including harmful or dependent drinking patterns, than their female counterparts [35]. Several factors predict risky alcohol use, including higher perceived stress, younger age, living alone, and increased reports of loneliness [7]. Consequently, structural conditions such as physical exercise and sports may be particularly advantageous for male students as they foster positive behavior and social support, potentially mitigating loneliness. However, among male students, participation in team sports is associated with more alcohol-related problems than participation in individual sports [13], underscoring the complexity of these relationships.

### Strengths and limitations

A key strength of this study is its nationwide sample of over 59,000 students, which provides robust statistical power and the ability to detect meaningful associations. Stratifying analyses by sex and adjusting for sociodemographic and behavioral factors enhance the robustness of the findings and offer nuanced insights into sex-specific associations. In addition, the use of validated self-report questionnaires to assess physical exercise [29, 30] and loneliness [24] supports the reliability and internal validity of the study.

However, several limitations need to be acknowledged. The cross-sectional design inherently limits causal interpretations, making it impossible to determine whether physical exercise reduces loneliness or whether individuals who are less lonely are more inclined to exercise. The relatively low response rate (35.1%) may have introduced selection bias by overrepresenting physically active or socially engaged students, as well as potentially vulnerable subgroups (e.g., those with mental health problems or loneliness) that could be either under- or overrepresented. Social stigma, particularly among males [49], may lead to underreporting of loneliness. The absence of social and structural characteristics in the exercise context may also restrict our understanding of how physical exercise influences loneliness [46]. Furthermore, while the T-ILS [24] is widely used, its brevity may not capture the full complexity of loneliness among students. Additionally, dichotomizing the T-ILS may obscure important gradations in loneliness. Self-reports of physical activity are susceptible to recall bias, social desirability, or item misunderstanding, potentially leading to over- or underestimation of activity levels [50]. Finally, unmeasured confounding factors such as personality traits, existing mental health conditions, and social media use may influence both exercise behaviors and feelings of loneliness.

### Considerations for further research

The optimal dose of physical exercise in terms of frequency, intensity, and duration associated with lower levels of loneliness among college and university students requires further investigation. Future studies should incorporate objective measures such as accelerometers and energy expenditure estimates for accurate exercise assessments [51]. Additionally, mixed-mode approaches can enhance response rates and provide richer data [52]. Longitudinal designs are necessary to understand the bidirectional relationship between physical activity patterns and loneliness to clarify potential causal mechanisms [20]. Finally, investigating the impact of relocation and adaptation to new environments may provide insights into students’ exercise patterns [53], thereby improving the reliability and applicability of findings.

The strong negative association between physical exercise and students’ experiences of loneliness identified in this study indicates that promoting physical exercise should be a relevant consideration for political, welfare, and educational institutions.

While exercise may alleviate loneliness among males and females, there is a lack of interventions and randomized controlled trials investigating this possible effect. To enhance participation, interventions should consider sex-specific needs, such as exercise preferences, within relevant structural and social contexts. Targeted interventions designed for physically inactive students or those who seldom engage in physical activity and report loneliness may be particularly beneficial.

## Conclusion

This study demonstrated that physical exercise was inversely associated with loneliness across frequency, intensity, and duration, among Norwegian college and university students. Associations were stronger among males than in females, particularly at higher exercise frequencies and longer durations. Taken together, these findings suggest that promoting physical exercise may be a relevant component of preventive initiatives targeting loneliness in higher education settings. Universities and welfare organizations should therefore consider supporting physical exercise programs for both sexes. Future research should prioritize sex-specific, longitudinal, and experimental studies to clarify causal relationships and examine possible long-term effects.

## Data Availability

Norwegian data protection regulations and GDPR impose restrictions on sharing of individual participant data. However, researchers may gain access to survey participant data by contacting the publication committee (borge.sivertsen@fhi.no). Approval from the Norwegian Regional Committee for Medical and Health Research Ethics (https://www.forskningsetikk.no/retningslinjer/med-helse/) is a pre-requirement for access to the data. The dataset is administrated by the NIPH, and guidelines for access to data are found at https://www.fhi.no/en/more/access-to-data.

## Abbreviations

AUDIT: Alcohol Use Disorders Identification Test
CI: Confidence interval
COVID-19: Coronavirus disease 2019OR—Odds Ratio
HUNT: Trøndelag Health Study
OR: Odds Ratio
Sammen: Student Welfare Organization in Bergen
SHoT: Student Health and Well-being Survey (Norway)
SiO: Student Welfare Organization in Oslo
SiT: Student Welfare Organization in Trondheim
SMS: Short Message Service
T-ILS: Three-Item Loneliness Scale
UCLA: UCLA Loneliness Scale (University of California, Los Ageles)
VIF: Variance inflation factor
